# Surgical airway procedures in emergency surgical patients: Results of what has become a back-up procedure

**DOI:** 10.1007/s00268-021-06110-7

**Published:** 2021-05-22

**Authors:** Gijs J. A. Willinge, Falco Hietbrink, Luke P. H. Leenen

**Affiliations:** 1 Department of Traumatology, University Medical Center Utrecht, Utrecht, The Netherlands; 2 Utrecht Trauma Center, G04. 228, Heidelberglaan 100, 3584 CX Utrecht, The Netherlands

## Abstract

**Background:**

Cricothyroidotomy and surgical tracheostomy are methods to secure airway patency. In emergency surgery, these methods are nowadays mostly reserved for patients unsuited for percutaneous procedures. Detailed description of complications and functional outcomes following both procedures is underreported in current literature. The aim of this study was to evaluate outcomes following cricothyroidotomy and tracheostomy in this presumed complex population.

**Methods:**

In this retrospective cohort study, adult emergency surgical patients treated with cricothyroidotomy and/or surgical tracheostomy were included. Postoperative complications and functional outcomes in trauma and non-trauma patients were evaluated.

**Results:**

Forty-one trauma patients and 11 non-trauma emergency surgical patients (mainly after elective onco-abdominal or vascular surgery) were included. Of 52 patients, seven underwent cricothyroidotomy pre-tracheostomy. Mortality was higher in non-trauma patients (*p* = 0.04) following both procedures. Over half of patients (56%, *n* = 29) regained unsupported airway patency with a tendency toward increased tracheostomy removal in trauma patients. Among complications, only pneumonia occurred frequently (60%, *n* = 31), with no relation to patient type. Other complications included local infection (5.8%, *n* = 4) and wound dehiscence (1.9%, *n* = 1). Adverse functional outcomes were frequently observed and were mild and self-limiting. Cervical spinal cord injury reduced overall unsupported airway patency (*p* = 0.01); with high cervical spinal cord injury related to adverse functional outcomes and increased home ventilation need.

**Conclusions:**

No major procedure-related complications or functional adverse events were encountered following cricothyroidotomy and surgical tracheostomy, even though only complex patients were included. Only mild, self-limiting functional problems occurred, especially in trauma patients with cervical injury who underwent early tracheostomy by longitudinal incision. This information can aid clinicians in making tailor-made decisions for individual patients.

## Introduction

Cricothyroidotomy and tracheostomy are both surgical methods to secure patency of the airway. Although serving the same purpose, both procedures are performed in different settings and have different indications. Cricothyroidotomy is performed in the emergency setting and provides an alternative method of gaining airway access when endotracheal intubation fails. Nowadays, with video-guided intubation techniques, this invasive procedure is rarely performed, but it can still be crucial in preventing anoxic encephalopathy and death. Considering the emergency setting, it is not surprising cricothyroidotomy is prone to cause severe short-term complications, including upper airway laceration, posterior tracheal perforation and nerve damage [1, 2]. Possibly due to its rare nature, knowledge about long-term outcomes following cricothyroidotomy is scarce.

Tracheostomy is a more common procedure in current medical practice [3]. Apart from its necessity during head&neck surgery, it is often electively performed when the need for prolonged mechanical ventilation (MV) is expected. A tracheostomy provides a way to maintain airway patency for an increased period of time. It has proved to cause less laryngeal complications, improve patient comfort and pulmonary hygiene, and reduce the use of sedation compared to prolonged endotracheal intubation [4–7]. Furthermore, it enables faster weaning from MV by reducing airway resistance and decreasing dead ventilation space [6, 8]. Two techniques for tracheostomy are currently used: percutaneous dilatational tracheostomy and surgical tracheostomy. The percutaneous method has gained preference over the surgical method in recent years due to suggested lower peri-operative complication rates and higher cost-effectiveness [9]. Surgical tracheostomy is now specifically used when contra-indications for percutaneous dilatational tracheostomy (such as history of neck surgery, unstable or immobile cervical spine, or anatomical anomalies) exist [10, 11]. Compared to cricothyroidotomy, tracheostomy causes less short-term complications. However, studies have described long-term complications and adverse functional outcomes following this procedure [12]. Although large database studies exist, detailed descriptions of these complications and functional outcomes are limited [13].

Improvement of detailed knowledge on consequences could assist surgeons in deciding whether to perform these invasive procedures, when to perform them and for which patient they would be beneficial. Therefore, the aim of this study was to analyze short- and long-term outcomes following both cricothyroidotomy and surgical tracheostomy in emergency surgical patients and to identify specific factors contributing to (relative) adverse outcomes.

## Materials and methods

### Study design and population

This was a single center, retrospective cohort study. A waiver of the Medical Ethical Committee of University Medical Center Utrecht (UMCU) was provided to conduct this study. Records of all emergency surgical patients receiving cricothyroidotomy and/or open surgical tracheostomy at the UMCU between January 2013 and December 2018 were identified. Emergency surgical patients aged ≥ 18 years at time of surgery, receiving cricothyroidotomy and/or surgical tracheostomy, were included. Patients were excluded if they received an initial airway procedure elsewhere (except pre-hospital cricothyroidotomy with presentation at our center subsequently). This study focused solely on the open surgical tracheostomy technique and not the percutaneous technique. Subsequently, as surgical tracheotomy is considered a back-up procedure at our hospital and is only used in patients with contra-indications for percutaneous tracheostomy, only complex emergency surgery patients for tracheostomy placement were included. For the remainder of this article, ‘tracheostomy’ will refer to the surgical technique.

### Study treatment specifics

Our center features an intermediate care unit (IMCU), which can provide hemodynamic monitoring and respiratory supportive care, and a fully equipped intensive care unit (ICU) [14]. Regarding cricothyroidotomy, standard procedure included conversion of cricothyroidotomy to tracheostomy within at least 14 days after cricothyroidotomy. This was generally done as soon as reasonably possible to avoid potential complications at the cricothyroidotomy surgery site, as recommended by current literature [15, 16]. For all tracheostomy procedures, one of two incision types was performed: a longitudinal incision or a hatch-shaped incision. Choice of incision type was based on personal preferences of the operating surgeon. All procedures were performed or supervised by senior specialist level trauma surgeons.

### Data collection

Relevant data were extracted from patient records. This included—but was not limited to—patient and injury characteristics, procedural characteristics, occurrence of postoperative complications at short- and long-term, physical airway-related complaints, surgical hardware-related complications, unsupported patency of airway, duration of weaning, duration of decuffing, time until removal of tracheostomy and mortality. Indications for tracheostomy were subdivided into three main groups: 1) physically threatened airway, 2) respiratory insufficient function (including ICU acquired weakness, neurological disability and prolonged/impaired weaning) and 3) alternative way of airway access (following cricothyroidotomy or endotracheal intubation complications). Included patients were divided into two groups for primary analysis: trauma patients and non-trauma patients. For trauma patients, injuries to the head/neck region were specified into head, brain, cervical osseous and cervical spinal cord injuries. Brain injury was additionally scored based on Glasgow Coma Scale (GCS) motor scores, with mild as GCS motor score 4–5 and severe as GCS motor score ≤ 3.

### Outcome measures

Primary outcome measures were airway-related complications at short- and long-term (up to a maximum of one year after the procedure) following both cricothyroidotomy and tracheostomy. These complications included lacerations of upper respiratory structures (such as vocal cords, pharynx and the tracheal wall), obstruction, posterior tracheal perforation, creation of false tracts, local infections, pneumothorax, wound dehiscence, pneumonia and mortality. Secondary outcomes were length of hospital stay, length of ICU stay, material-related complications and functional airway-related outcomes at short- and long-term follow-up (up to a maximum of one year after the procedure). This included phonation problems following tracheostomy, inability to autonomously clean airway, sputum management and swallowing dysfunction. Additionally, ability to wean, decuff and ultimately remove the tracheostomy was assessed. When applicable, duration of weaning, decuffing and time to removal of the tracheostomy was evaluated. To identify risk factors for adverse functional outcomes, patient and (if applicable) injury characteristics were analyzed.

### Statistical analysis

Statistical analysis was performed using IBM SPSS (Statistics for Windows, Version 25.0. Armonk, NY: IBM Corp). Descriptive data of continuous variables were summarized using appropriate measures of central tendency (i.e., mean, median) and dispersion (i.e., standard deviation, interquartile range [IQR]), depending on the distribution of variables. Categorical variables were presented using frequency measures. The Pearson Chi-square test and Fisher Exact test (two-sided) were used to analyze statistical significance of relations between categorical variables. The independent t-test was used for continuous variables. A p-value ≤ 0.05 indicated a statistically significant relation between variables.

## Results

**Fig. 1:**
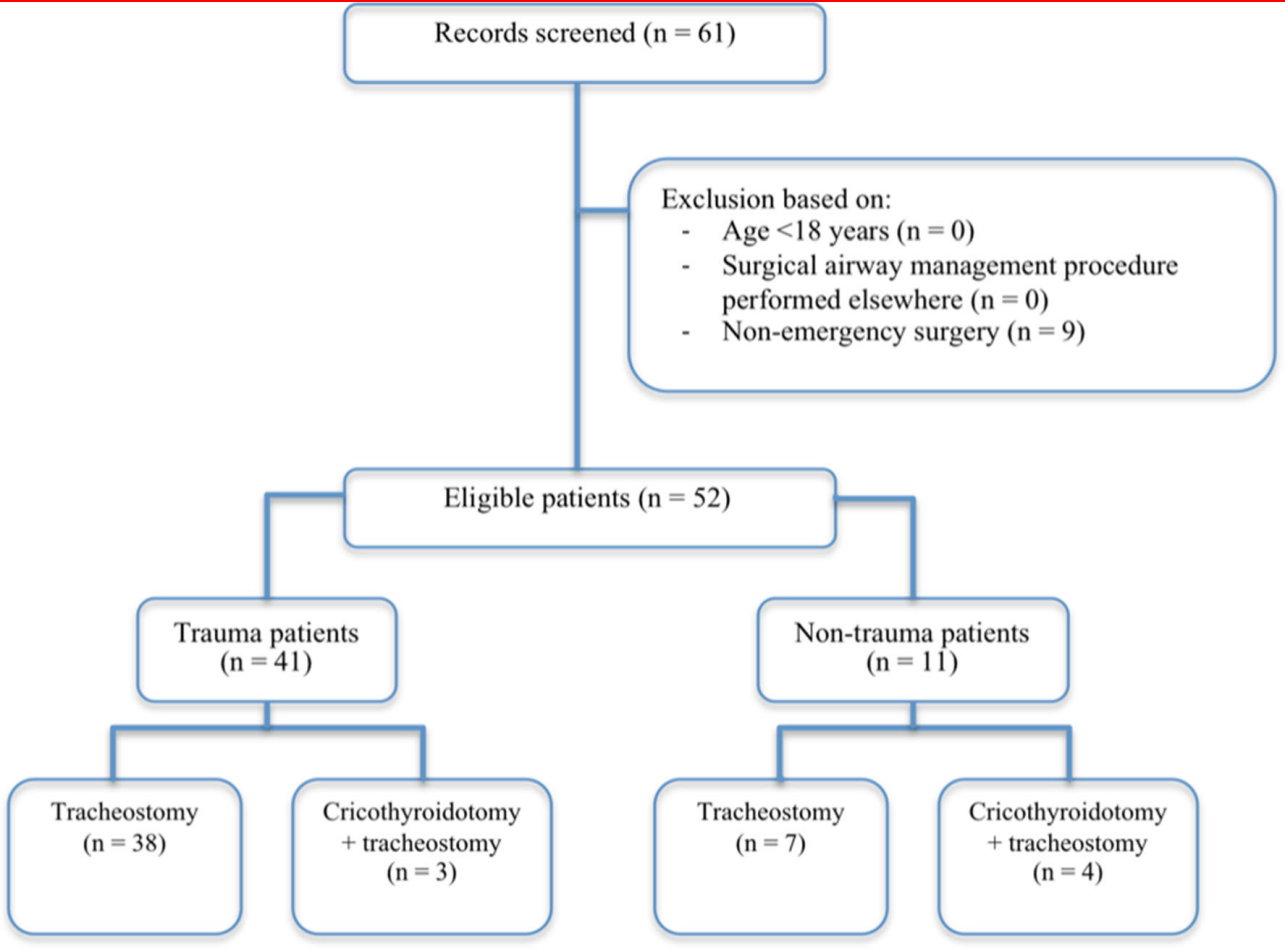
Flowchart of patient selection for study inclusion

A total of 61 patients received cricothyroidotomy and/or tracheostomy of which 52 met inclusion criteria (Fig. 1). Of these 52 patients, 45 underwent isolated tracheostomy and seven received cricothyroidotomy before tracheostomy. All cricothyroidotomies were converted into a tracheostomy within three days. Of all included patients, 41 (79%) were trauma patients. Median age was 54 years (IQR: 35) in the trauma group and 67 years (IQR: 13) in the non-trauma group, with a relatively low median age of cricothyroidotomy patients in both groups (Table 1). ASA score was higher in non-trauma patients compared to trauma patients. The non-trauma group consisted mainly of patients in need of surgical airway management after complications following elective abdominal or vascular surgery. Excessive sputum production pre-tracheostomy (reduced airway hygiene) was observed in 19 (37%) patients.

**Table 1:** Baseline characteristics

Variables	Tracheostomy		Cricothyroidotomy + tracheostomy	
	Trauma *N* = 38	Non-trauma *N* = 7	Trauma *N* = 3	Non-trauma *N* = 4
Demographics	Median (IQR)	Median (IQR)	Median (IQR)	Median (IQR)
Age (years)	55 (37)	67 (9)	39**	62 (24)
BMI	24 (9)	27 (6)	25**	28 (11)

Variables	Tracheostomy		Cricothyroidotomy + tracheostomy	
	Trauma *N* = 38	Non-trauma *N* = 7	Trauma *N* = 3	Non-trauma *N* = 4
	*N* (%)	*N* (%)	*N* (%)	*N* (%)
Male	25 (66)	4 (57)	3 (100)	1 (25)
Diabetes mellitus	4 (11)	1 (14)	0 (0)	0 (0)
ASA score				
1	0 (0)	0 (0)	0 (0)	0 (0)
2	23 (61)	1 (14)	2 (67)	0 (0)
3	15 (40)	6 (86)	1 (33)	3 (75)
4	0 (0)	0 (0)	0 (0)	1 (25)
Cervical characteristics				
Obesity	7 (18)	1 (14)	0 (0)	1 (25)
Anatomical abnormalities	4 (11)	0 (0)	0 (0)	0 (0)
Surgical history in cervical region	1 (3)	1 (14)	0 (0)	0 (0)
Pre-tracheostomy swallowing dysfunction	7 (18)	0 (0)	0 (0)	0 (0)
Excessive sputum pre-tracheostomy	15 (40)	3 (43)	0 (0)	1 (25)

Variables	Tracheostomy		Cricothyroidotomy + tracheostomy	
	Trauma *N* = 38	Non-trauma *N* = 7	Trauma *N* = 3	Non-trauma *N* = 4
Clinical characteristics	Median (IQR)	Median (IQR)	Median (IQR)	Median (IQR)
ISS*	27 (15)	–	22**	–
Vital signs at day of tracheostomy				
Systolic blood pressure	120 (35)	120 (38)	90**	132 (17)
Diastolic blood pressure	70 (20)	67 (30)	75**	64.8 (12)
Heart rate	93 (37)	87 (41)	90**	95 (34)

Variables	Tracheostomy		Cricothyroidotomy + tracheostomy	
	Trauma *N* = 38	Non-trauma *N* = 7	Trauma *N* = 3	Non-trauma *N* = 4
	*N* (%)	*N* (%)	*N* (%)	*N* (%)
ISS > 15*	32 (84)	–	2 (67)	–
GCS*				
GCS motor ≤ 3	18 (47)	1 (14)	1 (33)	2 (25)
GCS ≤ 8	20 (53)	1 (14)	1 (33)	2 (25)
ICU admission	38 (100)	6 (86)	3 (100)	3 (75
IMCU admission	0 (0)	1 (14)	0 (0)	1 (25)
Trauma injury specifics				
Head injury	23 (61)	0 (0)	1 (33)	0 (0)
Brain injury*				
Mild (GCS motor 4–5)	9 (24)	0 (0)	1 (33)	0 (0)
Severe (GCS motor ≤ 3)	18 (47)	0 (0)	0 (0)	0 (0)
Cervical osseous injury	19 (50)	0 (0)	2 (67)	0 (0)
Cervical spinal cord injury				
Level C1-C4	5 (13)	0 (0)	0 (0)	0 (0)
Level C5-C8	2 (5)	0 (0)	0 (0)	0 (0)
Procedural specifics				
Time from trauma/initial surgery to tracheostomy				
Early (≤ 14 days) tracheostomy	28 (74)	3 (43)	3 (100)	4 (100)
Late (> 14 days) tracheostomy	10 (27)	4 (57)	0 (0)	0 (0)
Indication for tracheostomy				
Physically threatened airway	3 (8)	0 (0)	1 (33)	1 (25)
Insufficient respiratory function	34 (90)	6 (86)	0 (0)	0 (0)
Alternative way of airway management	1 (3)	1 (14)	2 (67)	3 (75)
Incision type*				
Longitudinal	26 (69)	2 (29)	2 (67)	2 (50)
U-shaped	9 (24)	2 (29)	0 (0)	1 (25)

*IQR* Interquartile range, *BMI* Body Mass Index, *ASA* American society of Anesthesiologists, *ISS* Injury severity score, *GCS* Glasgow coma scale, *ICU* Intensive care unit, *IMCU* Intermediate care unit

*Missing data: ISS was missing in 8% (*n* = 3) of trauma tracheostomy patients. No ISS was scored in non-trauma patients. GCS was missing in 9% (*n* = 4) of total tracheostomy patients; GCS was missing in 5% (*n* = 2) of trauma tracheostomy patients and in 29% (*n* = 2) of non-trauma tracheostomy patients. GCS was missing in 2 of non-trauma cricothyroidotomy + tracheostomy patients. Incision type was missing in 15% (*n* = 7) of total tracheostomy patients; Incision type was missing in 8% (*n* = 3) of trauma tracheostomy patients and in 43% (*n* = 3) of non-trauma tracheostomy patients. Incision type was missing in 33% (*n* = 1) of trauma cricothyroidotomy + tracheostomy patients and in 25% (*n* = 1) of non-trauma cricothyroidotomy + tracheostomy patients

**In several cases, IQR could not be presented due to group size of *n* ≤ 3

Focusing on procedural specifics, prolonged insufficient respiratory function was the most common indication for tracheostomy and the longitudinal incision was most frequently performed. Indications for cricothyroidotomy included unsuccessful intubation (*n* = 5; 71%) and acute physically threatened airway (*n* = 2; 29%). Of the latter, one patient was successfully intubated after which the endotracheal tube was obstructed by active bleeding without successful exchange, and cricothyroidotomy was performed. The other patient was impossible to intubate due to facial injuries and therefore underwent primary cricothyroidotomy.

### Surgical outcomes

Mortality within one year after tracheostomy was relatively high in non-trauma patients, with 45% (*n* = 5) compared to 15% (*n* = 6) in trauma patients (*p* = 0.04) (Table 2). Our data showed no direct link between cricothyroidotomy and/or tracheostomy and the cause of death. For trauma patients, the primary cause of mortality was a direct consequence of the initial trauma, mainly neurological. For non-trauma patients, this was as a consequence of the underlying medical condition (e.g., sepsis, cancer, abdominal ischemia). Of trauma patients, 90% (*n* = 37) was successfully weaned from MV compared to 55% (*n* = 6) of non-trauma patients (*p* = 0.01). 59% of trauma patients (*n* = 24) regained unsupported airway patency following both cricothyroidotomy and tracheostomy, compared to 45% (*n* = 5) of non-trauma patients (*p* = 0.5). Length of ICU stay was longer in non-trauma patients (median 38 days, IQR 44) compared to trauma patients (median 16 days, IQR 13) (*p* = 0.05). Focusing on injury characteristics in trauma patients, cervical spinal cord injury was associated with decreased successful weaning rate (*p* = 0.01), decreased unsupported airway patency (*p* = 0.01) and less removal of tracheostomy (*p* = 0.01). No significant associations between these outcomes and other injury characteristics existed. Among complications, only pneumonia was frequently present in all patient groups (60%, *n* = 31), with no significant relation to specific patient type. Other procedure-related complications included local infection (5.8%, *n* = 4) and wound dehiscence (1.9%, *n* = 1).

**Table 2:** Comparison of outcomes between tracheostomy and cricothyroidotomy + tracheostomy in trauma and non-trauma patients

Variables	Tracheostomy		Cricothyroidotomy + tracheostomy	
	Trauma	Non-trauma	Trauma	Non-trauma
	*N* = 38	*N* = 7	*N* = 3	*N* = 4
	*N* (%)	*N* (%)	*N* (%)	*N* (%)
Complications				
Upper airway lacerations	0 (0)	0 (0)	0 (0)	0 (0)
Posterior tracheal perforation	0 (0)	0 (0)	0 (0)	0 (0)
False tracts	0 (0)	0 (0)	0 (0)	0 (0)
Pneumothorax	0 (0)	0 (0)	0 (0)	0 (0)
Wound dehiscence	1 (3)	0 (0)	0 (0)	0 (0)
Local infection	2 (5)	0 (0)	0 (0)	1 (25)
Pneumonia	24 (63)	3 (43)	1 (33)	3 (75)
Obstruction	1 (3)	0 (0)	0 (0)	0 (0)
Mortality < 1 year after tracheostomy	**6 (16)**	**4 (57)**	**0 (0)**	**1 (25)**
Tracheostomy outcomes				
Successfully weaned	**34 (90)**	**3 (43)**	**3 (100)**	**3 (75)**
Decuffing complete	26 (68)	2 (29)	2 (67)	3 (75)
Removal of tracheostoma	21(55)	2 (29)	3 (100)	3 (75)
Overall unsupported airway patency	21 (55)	2 (29)	3 (100)	3 (75)
< 30 days after tracheostomy	17 (45)	0 (0)	3 (100)	2 (50)
< 90 days after tracheostomy*	20 (53)	2 (29)	3 (100)	3 (75)
< 1 year after tracheostomy*	20 (53)	2 (29)	3 (100)	3 (75)
Home ventilation	3 (8)	0 (0)	0 (0)	2 (50)

Variables	Tracheostomy		Cricothyroidotomy + tracheostomy	
	Trauma	Non-trauma	Trauma	Non-trauma
	*N* = 38	*N* = 7	*N* = 3	*N* = 4
	Median (IQR)	Median (IQR)	Median (IQR)	Median (IQR)
Duration weaning period (days)	2 (3)	21**	1**	1**
Time from trauma/initial surgery to no MV (days)	15 (13)	36**	6**	4**
Duration decuffing period (days)	8 (6)	19**	3**	7**
Duration tracheostoma in situ (days)	19 (19)	69**	8**	15**
Length of hospital stay (days)	39 (17)	66 (43)	17**	36 (46)
Length of ICU stay (days)	**16 (15)**	**45 (47)**	6**	19**

Variables	Tracheostomy		Cricothyroidotomy + tracheostomy	
	Trauma	Non-trauma	Trauma	Non-trauma
	*N* = 38	*N* = 7	*N* = 3	*N* = 4
	*N* (%)	*N* (%)	*N* (%)	*N* (%)
Functional outcomes				
Phonation problems	3 (8)	0 (0)	0 (0)	1 (25)
Physical airway complaints				
Pain/irritation	3 (8)	0 (0)	0 (0)	1 (25)
Swallowing dysfunction	7 (18)	1 (14)	0 (0)	0 (0)
Excessive sputum post tracheostomy	24 (63)	1 (14)	0 (0)	3 (75)
Sputum leakage at tracheostomy site	9 (24)	0 (0)	0 (0)	1 (25)
Inability to clean airway	23 (61)	2 (29)	0 (0)	1 (25)
Material-related outcomes				
Material-related complications	10 (32)	0 (0)	1 (33)	0 (0)
Luxation cannula	2 (5)	0 (0)	0 (0)	0 (0)
Acquired cuff insufficiency	8 (21)	0 (0)	0 (0)	0 (0)

*ICU* Intensive care unit, *MV* mechanical ventilation, *IQR* Interquartile range

Bold indicates a significant difference *(p* ≤ 0.05) between trauma and non-trauma subgroups.

*Missing data: Data < 90 days after tracheostomy was missing in 5% (*n* = 2) of trauma and 14% (*n* = 1) of non-trauma tracheostomy patients. Data < 1 year after tracheostomy was missing in 8% (*n* = 3) of trauma and 14% (*n* = 1) of non-trauma tracheostomy patients

**In several cases, IQR could not be presented due to group size of *n* ≤ 3

### Functional outcomes

Physical airway complaints (pain/irritation and swallowing dysfunction) predominantly occurred in trauma patients. All complaints started within ten days after tracheostomy and disappeared within one year. Material-related complications were exclusively seen in trauma patients, with acquired cuff insufficiency being most common. It was unknown if these adverse functional outcomes (non-material-related) and complications were a direct consequence of the tracheostomy or were caused by trauma-related factors (such as neurological injuries or injuries to the maxillofacial or cervical region). Furthermore, spinal cord injury (level C1-C4) was significantly associated with phonation problems (*p* = 0.03) and inability to clean airway (*p* = 0.05) (Table 3). Need for home ventilation support was also higher in these patients (*p* < 0.01). Of five patients with phonation problems after high cervical spinal cord injury, two had physical complaints during phonation after tracheostomy and three were unable to speak due to the nature of their injury.

**Table 3:** Evaluation of patient/injury/disease/procedural characteristics and functional outcomes

			Phonation problems *N* = 4								Physical airway complaints *N* = 12								Excessive sputum post-tracheostomy *N* = 28								Sputum leakage *N* = 10							Inability to clean airway *N* = 26						
			Trauma *N* = 3 (75%)				Non-trauma *N* = 1 (25%)				Trauma *N* = 10 (83%)				Non-trauma *N* = 2 (17%)				Trauma *N* = 24 (86%)				Non-trauma *N* = 4 (14%)				Trauma *N* = 9 (90%)			Non-trauma *N* = 1 (10%)				Trauma *N* = 23 (88%)					Non-trauma *N* = 3 (11%)	
			*N* (%)				*N* (%)				*N* (%)				*N* (%)				*N* (%)				*N* (%)				*N* (%)			*N* (%)				*N* (%)					*N* (%)	
Demographics																																								
Male			3 (75)				0 (0)				8 (67)				1 (8)				16 (57)				1 (4)				8 (80)			0 (0)				17 (65)					2 (8)	
Female			0 (0)				1 (25)				2 (17)				1 (8)				8 (29)				3 (11)				1 (10)			1 (10)				6 (23					1 (4)	
Diabetes			0 (0)				0 (0)				0 (0)				0 (0)				2 (7)				0 (0)				1 (10)			0 (0)				1 (4)					0 (0)	
ASA score																																								
1			0 (0)				0 (0)				0 (0)				0 (0)				0 (0)				0 (0)				0 (0)			0 (0)				0 (0)					0 (0)	
2			2 (50)				0 (0)				4 (33)				0 (0)				13 (46)				0 (0)				5 (50)			0 (0)				13 (50)					0 (0)	
3			1 (25)				1 (25)				6 (50)				2 (17)				11 (39)				4 (4)				4 (40)			1 (10)				10 (38)					2 (8)	
4			0 (0)				0 (0)				0 (0)				0 (0)				0 (0)				0 (0)				0 (0)			0 (0)				0 (0)					1 (4)	
Clinical characteristics																																								
GCS motor ≤ 3*			2 (50)				0 (0)				3 (25)				1 (8)				12 (43)				1 (4)				3 (30)			1 (10)				10 (38)					0 (0)	
GCS ≤ 8*			2 (50)				0 (0)				4 (33)				1 (8)				14 (50)				1 (4)				4 (40)			1 (10)				12 (46)					0 (0)	
Spine immobilisation at time of tracheostomy			2 (50)				0 (0)				8 (67)				0 (0)				18 (64)				0 (0)				7 (70)			0 (0)				18 (69)					0 (0)	
ICU admission			0 (0)				1 (25)				10 (83)				1 (8)				24 (86)				3 (11)				9 (90)			0 (0)				23 (82)					3 (12)	
Excessive sputum production pre-tracheostomy			1 (25)				0 (0)				6 (50)				0 (0)				11 (39)				2 (50)				4 (40)			0 (0)				**13 (50)**					2 (67)	
Trauma injury specifics																																								
Head injury			0 (0)				0 (0)				6 (50)				0 (0)				15 (54)				0 (0)				4 (40)			0 (0)				13 (50)					0 (0)	
Brain injury**																																								
Mild (GCS motor 4–5)			0 (0)				0 (0)				3 (25)				0 (0)				7 (25)				0 (0)				3 (30)			0 (0)				7 (27)					0 (0)	
Severe (GCS motor ≤ 3)			2 (50)				0 (0)				3 (25)				0 (0)				12 (43)				0 (0)				3 (30)			0 (0)				10 (38)					0 (0)	
Cervical injury			3 (75)				0 (0)				5 (42)				0 (0)				13 (46)				0 (0)				6 (60)			0 (0)				13 (50)					0 (0)	
Cervical spinal cord injury																																								
Level C1-C4			**2 (50)**				0 (0)				1 (8)				0 (0)				3 (11)				0 (0)				2 (20)			0 (0)				**5 (19)**					0 (0)	
Level C5-C8			0 (0)				0 (0)				1 (8)				0 (0)				2 (7)				0 (0)				0 (0)			0 (0)				2 (8)					0 (0)	
ISS > 15			3 (75)				–				8 (67)				–				19 (68)				–				8 (80)			–				2 (8)					–	
Procedural specifics																																								
Time to tracheostomy																																								
Early (≤ 14 days)			3 (75)				1 (25)				8 (67)				1 (8)				17 (61)				4 (14)				8 (80)			1 (10)				21 (81)					2 (8)	
Late (> 14 days)			0 (0)				0 (0)				2 (17)				1 (8)				7 (25)				0 (0)				1 (10)			0 (0)				2 (8)					1 (4)	
Indication																																								
Physically threatened airway			0 (0)				1 (25)				0 (0)				0 (0)				1 (4)				1 (4)				1 (10)			0 (0)				1 (4)					1 (4)	
Insufficient respiratory function			3 (75)				0 (0)				9 (75)				1 (8)				22 (79)				1 (4)				8 (80)			0 (0)				22 (85)					2 (8)	
Alternative way of airway access			0 (0)				0 (0)				1 (8)				1 (8				1 (4)				2 (7)				0 (0)			1 (10)				0 (0)					0 (0)	
Incision type																																								
Longitudinal			3 (75)				0 (0)				7 (58)				1 (8)				19 (68)				1 (4)				**9 (90)**			1 (10)				7 (27)					0 (0)	
U-shaped			0 (0)				1 (25)				3 (25)				0 (0)				5 (18)				2 (7)				**0 (0)**			0 (0)				7 (27)					2 (8)	
Cricothyroidotomy pre-tracheostomy			0 (0)				1 (25)				0 (0)				1 (8)				0 (0)				3 (11)				0 (0)			1 (10)				0 (0)					1 (4)	

*ASA* American society of anesthesiologists, *GCS* Glasgow coma scale

Bold indicates a significant relation between variables with *p* ≤ 0.05

*Missing data: GCS was missing in one non-trauma patient, incision type was missing in one non-trauma patient

**Brain injury was scored as mild if GCS motor 3–5, and scored as severe if GCS motor ≤ 3

For trauma patients, a significant relation existed between incision type and increased sputum leakage at tracheostomy site (*p* = 0.04). Furthermore, of patients with increased sputum leakage, 80% underwent early tracheostomy (≤ 14 days) and 60% had cervical injuries. There was no significant relation between sputum leakage after tracheostomy and inability to clean airway, or excessive sputum pre-tracheostomy. Inability to clean airway was more frequently seen in patients receiving early tracheostomy (≤ 14 days after trauma/initial surgery).

## Discussion

This study provided a detailed description of short- and long-term clinical outcomes following surgical airway procedures in emergency surgical patients, in a time where these procedures are reserved for patients unsuited for percutaneous procedures. Over half of patients regained unsupported airway patency, with a tendency toward increased removal of tracheostomy in trauma patients. As expected, cervical spinal cord injury was associated with reduced unsupported patency of airway. No procedure-related complications were encountered. Adverse functional outcomes did occur, but were considered mild and were all self-limiting.

Mortality rate after tracheostomy is high, emphasizing the dire situation of patients in need of cricothyroidotomy and/or tracheostomy [17, 18]. In our study, overall mortality was 21%, with mortality in non-trauma patients (45%) even three times higher compared to trauma patients (15%). This is possibly due to the medical condition of non-trauma patients before tracheostomy (as represented by ASA scores at baseline) and underlying disease, which initially caused their ICU admission. No direct link was found between surgical airway management procedures and cause of death. Patients predominantly died due to direct consequences of trauma (e.g., neurological damage) or due to complications related to the underlying medical condition/disease (e.g., abdominal ischemia, sepsis, cancer, complication of aortic repair). Although no link between surgical airway management and mortality existed, it can be stated that the sole need for surgical airway management, especially in non-trauma patients, is a harbinger of complications. Other studies similarly showed a higher mortality rate in patients with underlying (specifically respiratory) disease [19, 20]. Additionally, regarding length of ICU stay, it can be argued that non-trauma patients were more dependent of other ICU utilities besides respiratory support due to underlying disease, elongating their ICU stay. Mehta et al., with a sample size of 18.000 in the US, also showed improved outcomes following tracheostomy in trauma patients compared to non-trauma patients [21].

Although limited in number, cricothyroidotomy did not evidently cause short- or long-term complications—remarkable, since this is always done in urgent, life-threatening situations. This contradicts previous studies reporting several complications following cricothyroidotomy (e.g., cartilage injury, subglottic stenosis and phonation problems) [16, 22, 23]. Focusing on indications for cricothyroidotomy, impossibility to intubate due to facial/airway injuries is reported as one of the major indications 24. In our study, only one patient underwent cricothyroidotomy due to facial injuries. With approximately 350 polytrauma patients in our center per year, this relates to one cricothyroidotomy due to facial injuries per 1,750 polytrauma patients over a five-year period [25]. Thus, in a mature trauma center, cricothyroidotomy is becoming extremely rare in the ER and is more often performed outside the ER or in non-trauma settings.

Regarding complications following tracheostomy, only pneumonia was frequently present, with similar rates as reported by previous studies [13, 26]. No other procedural complications occurred, demonstrating the safety of tracheostomy as an airway patency management procedure, even in presumed complex patients. Material-related complications and adverse functional outcomes were observed more frequently, especially in trauma patients, which is in line with the recent literature [27]. However, as also shown by Silvester et al., trauma-related characteristics (such as injuries sustained during trauma or neck immobilization) may have influenced this Silvester et al. [28]. It can also be argued that trauma patients were more alert to these adverse functional outcomes, since they generally recovered more quickly. In our study, cervical spinal cord injuries significantly increased phonation problems, inability to clean airway and need for home ventilation support. The relation between excessive sputum production pre-tracheostomy and inability to clean airway (reduced airway hygiene) after tracheostomy indicates the problem was already present before tracheostomy and was possibly even part of its indication.

Evaluation of procedural specifics showed an association between a longitudinal incision and increased sputum leakage at tracheostomy site. Although sputum leakage was only significantly associated with a longitudinal incision, our results suggest a combination of cervical injury, early tracheostomy and a longitudinal incision was most likely to cause sputum leakage. This may be explained by local swelling of the incision site at time of tracheostomy, with sputum leakage occurring after reduction in swelling. Caregivers should consider variables such as injury type (e.g., cervical (spinal cord) injury with regional swelling), timing of tracheostomy and incision type when applying surgical airway management care.

## Strengths and limitations

This study provided a detailed description of tracheostomy outcomes—including functional and daily tracheostomy care-related outcomes—in a time where this procedure is predominantly reserved as a back-up procedure. Furthermore, these results could be related to patient, injury/disease and procedural characteristics, thus enhancing specificity.

Our study was limited by a relatively small sample size and subsequently, the inability to perform adequate multivariate analysis to control for potential confounders. The small sample size of surgical tracheostomy technique in emergency surgery patients was primarily caused by the increasing rarity of tracheostomy procedures and the recent shift to the percutaneous tracheostomy technique in current medical practice. Another limitation was its retrospective nature. Furthermore, in our study, no validated patient reported outcome measures were used to analyze functional outcomes. Finally, outcomes for patients who were spared tracheostomy by postponing the procedure were not accounted for. These outcomes could affect decision-making regarding both indication and timing.

## Conclusion

In an era in which surgical airway management has become a back-up procedure and is reserved for complex patients only, a detailed description of clinical results is scarce. In our study, all patients had either a cervical or systemic problem, which posed a contra-indication for percutaneous procedures. Nevertheless, no major procedure-related complications or functional adverse events were encountered; only temporary functional complications occurred. Our results show that trauma patients with cervical injury, who underwent early tracheostomy with a longitudinal incision, were especially at risk of these temporary complications. This information can aid clinicians in making tailor-made decisions for individual patients.

## Data Availability

The datasets used and/or analyzed during the current study are available from the corresponding author on reasonable request.
